# Predicting Lymph Node Metastasis in Endometrial Cancer Using Serum CA125 Combined with Immunohistochemical Markers PR and Ki67, and a Comparison with Other Prediction Models

**DOI:** 10.1371/journal.pone.0155145

**Published:** 2016-05-10

**Authors:** Bingyi Yang, Boer Shan, Xiaohong Xue, Huaying Wang, Weiwei Shan, Chengcheng Ning, Qiongjie Zhou, Xiaojun Chen, Xuezhen Luo

**Affiliations:** 1 Department of Gynecology, Obstetrics and Gynecology Hospital of Fudan University, Shanghai 200011, China; 2 Shanghai Key Laboratory of Female Reproductive Endocrine Related Diseases, Shanghai 200011, China; 3 Fudan University Shanghai Cancer Center, Shanghai 200032, China; H. Lee Moffitt Cancer Center & Research Institute, UNITED STATES

## Abstract

We aimed to evaluate the value of immunohistochemical markers and serum CA125 in predicting the risk of lymph node metastasis (LNM) in women with endometrial cancer and to identify a low-risk group of LNM. The medical records of 370 patients with endometrial endometrioid adenocarcinoma who underwent surgical staging in the Obstetrics & Gynecology Hospital of Fudan University were collected and retrospectively reviewed. Immunohistochemical markers were screened. A model using serum cancer antigen 125 (CA125) level, the immunohistochemical markers progesterone receptor (PR) and Ki67 was created for prediction of LNM. A predicted probability of 4% among these patients was defined as low risk. The developed model was externally validated in 200 patients from Shanghai Cancer Center. The efficiency of the model was compared with three other reported prediction models. Patients with serum CA125 < 30.0 IU/mL, either or both of positive PR staining > 50% and Ki67 < 40% in cancer lesion were defined as low risk for LNM. The model showed good discrimination with an area under the receiver operating characteristic curve of 0.82. The model classified 61.9% (229/370) of patients as being at low risk for LNM. Among these 229 patients, 6 patients (2.6%) had LNM and the negative predictive value was 97.4% (223/229). The sensitivity and specificity of the model were 84.6% and 67.4% respectively. In the validation cohort, the model classified 59.5% (119/200) of patients as low-risk, 3 out of these 119 patients (2.5%) has LNM. Our model showed a predictive power similar to those of two previously reported prediction models. The prediction model using serum CA125 and the immunohistochemical markers PR and Ki67 is useful to predict patients with a low risk of LNM and has the potential to provide valuable guidance to clinicians in the treatment of patients with endometrioid endometrial cancer.

## Introduction

Lymph node metastasis (LNM) is one of the most important prognostic factors in endometrial cancer [1,2]. Although lymphadenectomy is the best way to identify LNM, its clinical value remains controversial [3–6]. Two randomized trials suggested no survival benefit for routine lymphadenectomy in endometrial cancer [3,4]. It is thought that the risk of lymphadenectomy outweighs its benefit in low-risk endometrial cancer patients [5,6]. On the other hand, the prognosis of the patients with LNM would be poor if LNM was not identified and these patients received only hysterectomy without lymphadenectomy or post-operative radiotherapy. Thus, in order to provide endometrial cancer patients with precise and appropriate treatment, it is important to find ways to correctly predict patients with low-risk of LNM either before surgery or after hysterectomy without lymphadenectomy.

Reported studies have used various factors to predict LNM [7–11], such as magnetic resonance imaging (MRI) combined with cancer antigen 125 (CA125); tumor size with myometrial invasion and histological type and grade; and lymphovascular space involvement (LVSI) with immunostaining of estrogen receptor (ER) and progesterone receptor (PR) in endometrial lesion.

In this study, we tried to build a new predicting model using the combination of immunuhistochemical markers and serum CA125 which are somehow objective, cheap and easily accessible in clinical work. After screening commonly used immunohistochemical markers in endometrial cancer lesions, we selected PR and Ki67, in combination with serum CA125 level to build a model to predict the risk of LNM in endometrial cancer. The efficiency of the model was compared with those of three other reported prediction models [7–10].

## Methods

### Patient selection

**Fig 1:**
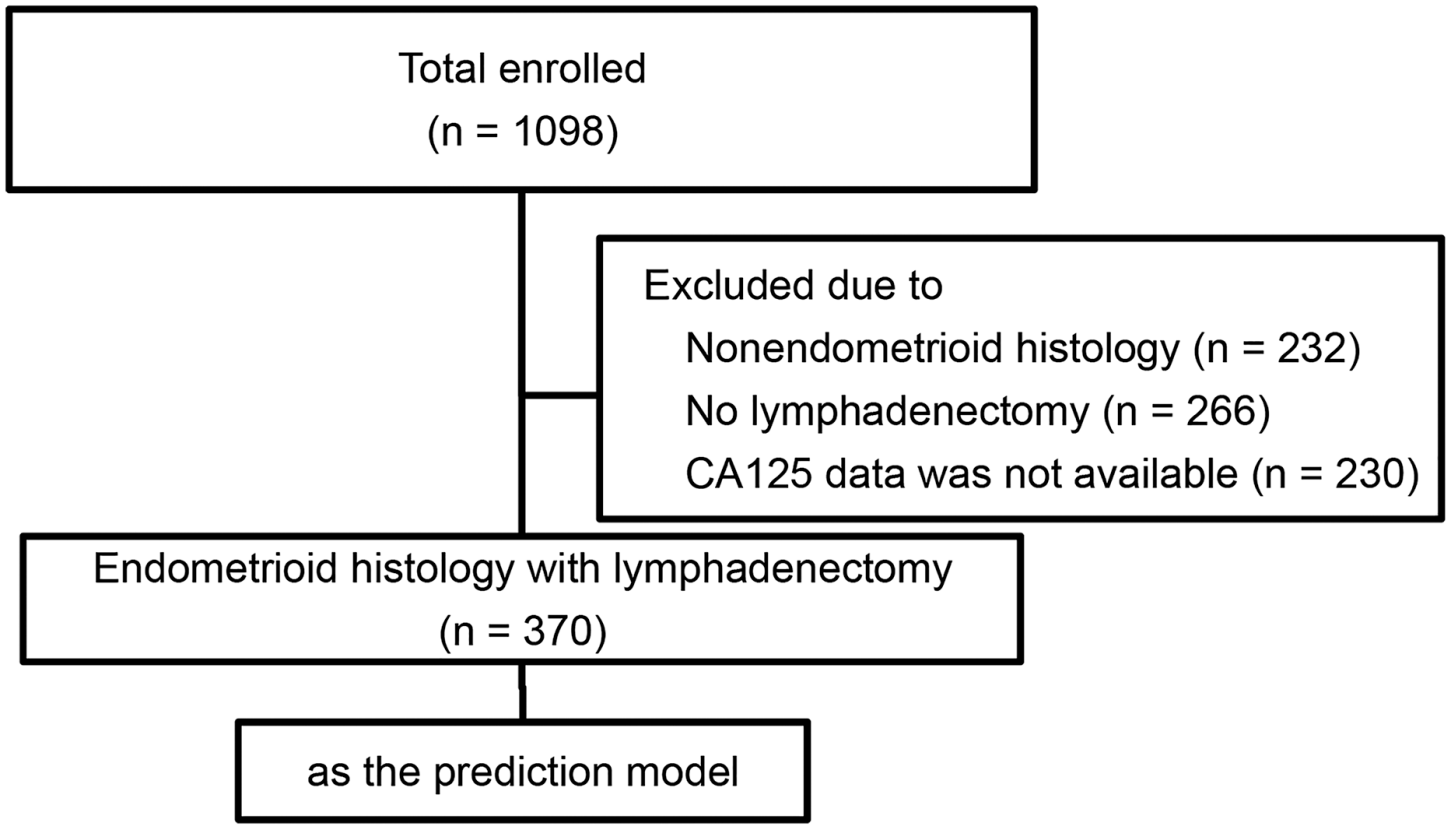
Flowchart of selection of patients in prediction cohort.

This descriptive study was based on a retrospective review of records of patients diagnosed with endometrial endometrioid adenocarcinoma. The research protocol was approved by the Ethics Committee of the Obstetrics & Gynecology Hospital of Fudan University (Ob&Gyn Hospital) and Fudan University Shanghai Cancer Center. All patients signed informed consent. All cases were re-evaluated and classified according to the World Health Organization (WHO) pathological classification (2014). Patients from Ob&Gyn Hospital were used to construct the prediction model. Another group of patients from Shanghai Cancer Center were collected as external validation cohort. Between January 2009 and April 2014, a total of 1098 endometrial cancer patients were treated in Ob&Gyn Hospital. Among these patients, those with endometrioid histological subtype who underwent comprehensive surgical staging with pelvic lymphadenectomy, having available preoperative serum CA125 levels and postoperative immunohistochemical staining of ER, PR, Ki67 and p53 were enrolled in the study. Patients with non-endometrioid histological subtypes or those with incomplete medical record as needed above were excluded. Comprehensive surgical staging included total hysterectomy, bilateral salpingo-oophorectomy, washing cytology, and systemic pelvic lymph node dissection. There were no restrictions on para-aortic lymphadenectomy. Eventually 370 patients from Ob&Gyn Hospital were enrolled in the study (Fig 1). 200 endometrial cancer patients treated in Shanghai Cancer Center from 2009 to 2014 who meet the inclusion criteria mentioned above were randomly selected as the validation group.

### Biomarkers and immunohistochemistry

ER, PR, Ki67 and p53 were evaluated in endometrial cancer lesions after hysterectomy using immunohistochemical staining. Surgical samples were processed as described [12]. Briefly, samples were cut into 0.5 cm slices, fixed immediately in formalin (10%) and then embedded in paraffin. Serial 3 μm thick coronal sections were cut from paraffin blocks and attached to slides with Vectabond^™^ (Vector Laboratories, Fisher Scientific, Pittsburgh, PA, USA). To ensure uniform handling of samples, all sections were processed with the same standard in the department of Pathology. Immunohistochemical studies were carried out with the *IHC Protocol F Program of Leica BOND-MAX^™^ Detection System (Leica) according to the manufacturer’s instruction. Tissue slides were dried overnight at 70°C, dewaxed with xylene, and gradually hydrated. Sections were subjected to heat-induced epitope retrieval for 20 min at 100°C, cooled to 20°C and then treated with 0.3% H_2_O_2_ in methanol for 5 min to block endogenous peroxidase activity. The following primary mouse monoclonal antibodies: ER (clone EP1, 1:150), PR (clone PgR636, 1:500), ki67 (clone MIB-1, 1:300) and p53 (clone DO-7, 1:200) (all purchased from DakoCytomation, Glostrup, Denmark) were applied separately and incubated overnight at 4°C. Samples were incubated for 30 min at room temperature by using an anti-mouse secondary antibody (Leica). Diaminobenzidine was used as a chromogen (DAB Substrate System, DAKO). Sections were counterstained with hematoxylin, dehydrated, and mounted. PBS (phosphate-buffered saline) and 1% Tween 20 were used as wash buffer for three times between each two procedures. Negative controls were subjected to the same procedure, except that the primary antibody was replaced by PBS. A section on each case was examined microscopically to confirm the diagnosis by two independent pathologists (ZQ and WL). For myometrial invasion evaluation, we measured the depth of tumor invasion in the deepest invasion area of myometrium. The thickness of the uterine wall at the same point was also measured. If the depth of myometrial invasion was one-half or more of the myometrium, deep myometrial invasion was defined. If the diagnosis of the two observers differed, a seminar would be held to discuss the case and a final decision would be made in the department of pathology.

Quantification of positive staining for ER, PR, Ki67 and p53 in nuclei of histologically identified neoplastic cells was calculated. Without acknowledgement of the clinical pathological parameters, two independent observers (WC & TX) evaluated 100 tumor cells in each field and calculated the average positive percentages of each marker in 5 adjacent field of view (with a × 40 objective) in the same invasive portion of the most active tumor areas. If the counts between two observers differed by more than 10%, the count was repeated with a multi-head microscope and a consensus was obtained. The number of cases with counts between two observers more than 10% were 15, 8, 40 and 13 for ER, PR, Ki67 and p53 respectively. There was no case of which the scoring by the two observers differed more than 20%.

Serum CA125 level was analyzed within one week before surgery and determined by radioimmunoassay (Modular E170 analyzer, Roche Diagnostics, Indianapolis, IN, USA).

### Three reported prediction models

In order to evaluate the predictive efficiency of our model, we compared our prediction model with three reported prediction models for LNM. These three models are: 1) the Mayo clinic model suggested by Mariani et al. (Model A) [7,8]; 2) a model modified from the Korean Gynecologic Oncology Group (KGOG) study suggested by Kang et al. (Model B) [9]; and 3) a model modified from a recursive partitioning (RP) model suggested by Ballester et al. (Model C) [10]. Detailed descriptions of these models are summarized in Table 1.

**Table 1:** Description of three reported prediction models for lymph node metastasis (LNM) in endometrial cancer.

Model	Criteria for low risk of LNM
**Model A** [7, 8]	Intraoperative frozen pathological grade 1–2, myometrial invasion ≤ 50%
	Primary tumor diameter ≤ 2 cm
	Endometrioid histology
**Model B** [9]	MRI shows no deep myometrial invasion, enlarged lymph nodes or extension beyond uterine corpus
	CA125 less than 35 IU/mL
	Endometrioid histology
**Model C** [10]	For FIGO stage IA grade 1 or 2: 1) ER ≥ 30%; 2) ER < 30% and PR ≥ 15%. For FIGO stage IA grade 3, or FIGO stage IB grade 1 or 2: 1) no LVSI; 2) LVSI and PR ≥ 15%
	Endometrioid histology

### Statistical Analysis

The correlation between semi-quantitative immunostaining of ER, PR, Ki67 and p53 as well as serum CA125 level and lymph nodal status was evaluated using the Chi-square test, and the optimal cutoff for each parameter was determined by Youden’s index. The cutoff with the biggest Youden’s index was chosen as the optimal cutoff.

Continuous variables were characterized using the median and range, and categorical variables were characterized using frequency and percentages.

By using univariate and multivariate logistic regression models, a prediction model was developed. The discrimination performance of this model was determined by calculating the area under the receiver operating characteristic curve (AUC). The calibration of this model was determined by the Hosmer-Lemeshow goodness-of-fit test. We validated the model internally using a bootstrap method, which is based on a re-sampling obtained by randomly drawing replacements from the original data set (1000 re-samplings were performed).

Before analyzing this model, the study steering committee determined a cutoff of predicted probability of 4% to identify a low-risk group. This cutoff of 4% has previously been supported as a negligible risk [9,13]. The negative predictive value was also calculated. The negative likelihood ratio ([1-sensitivity]/specificity) of the predicted low-risk group was calculated [14]. Sensitivity was calculated as the proportion of non-low-risk patients among the patients with LNM. Specificity was calculated as the proportion of low-risk patients among the patients without LNM. All statistical analyses were performed using STATA, version 11.0 (STATA, College Station, TX, USA). A p- value less than 0.05 was considered statistically significant.

## Results

### General characteristics of patients

Basic characteristics of the prediction cohort and validation cohort were seen in Table 2. No significant difference was found between the two cohorts. To prevent from selection bias of the 370 patients (prediction cohort) selected from the original 1098 patients (among which 866 patients were endometrioid adenocarcinoma) in Ob&Gyn Hospital, we also compared the basic characteristics of the selected 370 patients with the 866 endometrioid adenocarcinoma cases from the original 1098 patients. No significant difference was found in patients suffering from endometrioid endometrial cancer between the two groups (Table 3). We did not compare FIGO staging between these two groups because 266 patients out of the 866 patients did not receive lymphadenectomy.

**Table 2:** Basic characteristics of prediction cohort and validation cohort.

Characteristics	Prediction Cohort		Validation Cohort		P value
Number of patients	370	%	200	%	
**Age at diagnosis (y)**					0.84
**Median (mean)**	55		54		
**Range**	21–78		24–78		
**FIGO stage (2014)**					0.52
**IA**	227	61.4	117	58.5	
**IB**	52	14.1	24	12.0	
**II**	36	9.7	20	10.0	
**III-IV**	55	14.9	39	19.5	
**Grade (preoperative biopsy)**					/
**1**	311	84.4	/		
**2**	18	4.4	/		
**3**	27	7.4	/		
**Atypical hyperplasia**	8	2.2	/		
**Without preoperative biopsies**	6	1.5	/		
**Grade (final pathology)**					0.10
**1**	229	61.9	107	53.5	
**2**	84	21.9	61	30.5	
**3**	57	16.3	32	16.0	
**Myometrial invasion**					0.86
**< 1/2**	262	69.7	143	71.5	
**≥ 1/2**	108	30.3	57	38.5	
**LVSI**					0.61
**Yes**	75	19.6	37	22.0	
**No**	295	80.4	163	78.0	
**Primary tumor diameter**					/
**≤ 2 cm**	135	34.4	/	/	
**> 2 cm**	235	65.6	/	/	
**Menopausal status**					0.73
**Pre-menopausal**	148	38.1	77	38.5	
**Post-menopausal**	222	61.9	123	61.5	
**Hypertension**	100	26.7	47	23.5	0.36
**Diabetes mellitus**	23	6.3	16	8.0	0.36
**Para-aortic node dissection**	89	23.7	31	15.5	0.42
**Node metastasis**	39	10.7	26	13.0	0.38
**Extent of lymphadenectomy**					0.87
**Median (mean)**	20		20		
**Range**	7–56		7–45		

**Table 3:** Basic characteristics of selected 370 patients and the 866 patients with endometrioid histology from original 1098 patients.

Characteristics	Selected 370 patients		Original 866 patients		P value
Number of patients	370	%	866	%	
**Age at diagnosis (y)**					0.27
**Median (mean)**	54		55		
**Range**	21–78		21–79		
**Grade (final pathology)**					0.52
**1**	229	61.9	542	62.6	
**2**	84	21.9	199	23.0	
**3**	57	16.3	125	14.4	
**Myometrial invasion**					
**< 1/2**	262	69.7	602	69.5	0.65
**≥ 1/2**	108	30.3	264	30.5	
**Menopausal status**					0.24
**Pre-menopausal**	148	38.1	378	43.6	
**Post-menopausal**	222	61.9	488	56.4	
**Hypertension**	100	26.7	216	24.9	0.44
**Diabetes mellitus**	23	6.3	65	7.5	0.42

### Screening predictors and constructing prediction model

**Fig 2:**
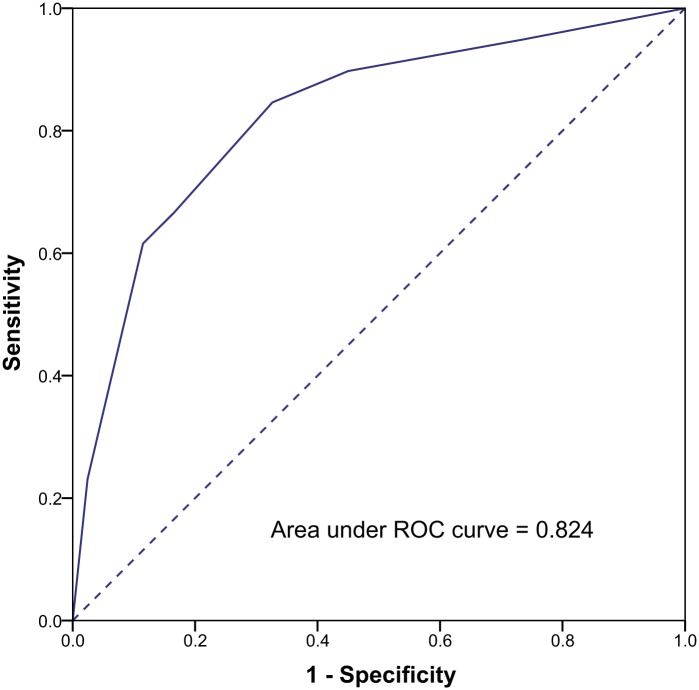
Performance of the prediction model for LNM in endometrial endometrioid cancer.

We selected commonly used immunohistochemical markers ER, PR, Ki67 and p53 as candidate predictors and screened these markers in the prediction cohort. Because a growing number of reports suggest that serum CA125 level correlates with the prognosis of endometrial cancer [15–17], and this variable is also easily accessed in clinical work, serum CA125 level was included in the screening as well. Univariate and multivariate logistic regression analyses were performed to screen candidate predictors (Table 4). All the five candidates (ER, PR, Ki67, p53 and CA125) were correlated with LNM in univariate analyses. Further internal validation was performed with multivariate logistic regression analyses. ER and p53 were not found to be correlated with LNM in multivariate logistic regression analyses, therefore these two candidates were excluded in the model. CA125, PR and Ki67 continued to show statistically significant correlations with LNM in multivariate logistic regression analyses and were selected to construct prediction model. The corresponding optimal cutoff for CA125 was 30.0 IU/mL and 50% for PR, 40% for Ki67. The AUC was calculated by using the predicted probability of nodal metastasis. As shown in Fig 2 the AUC was 0.82 (95% confidence interval [CI], 0.75–0.90). The low-risk group was defined by a predicted probability of nodal metastasis with a predefined cutoff of 4%. When we verified the low-risk group predicted by the model, we found that this group could be characterized as follows: serum CA125 < 30.0 IU/mL, tumor with either or both of positive PR staining > 50% and Ki67 < 40%. Therefore, we defined the low-risk group for nodal metastasis as patients who demonstrated the following clinical features: serum CA125 < 30.0 IU/mL, tumor with either or both of positive PR staining > 50% and Ki67 < 40%. Among the 370 patients in the prediction cohort, the model classified 229 patients (61.9%) as being at low risk. Among these 229 patients, 6 patients (2.6%) had LNM and the negative predictive value was 97.4% (223/229). 33 patients (23.4%) out of the remaining 141 non-low-risk patients had LNM. The sensitivity and specificity of the model were 84.6% and 67.4% respectively.

**Table 4:** Results of univariate and multivariate logistic regression analyses in the prediction cohort.

Variable	n	Univariate Coefficient	P	Multivariate Coefficient	P	Multivariate Coefficient	P	BootstrappedP	AUC
**p53 (> 25%)**	22 (6.0%)	1.275	0.013	0.806	0.191				0.553
**ER (< 25%)**	101 (27.3%)	0.821	0.018	0.555	0.203				0.591
**PR (< 50%)**	132 (48.9%)	0.956	0.005	0.858	0.047	1.159	0.003	0.001	0.616
**Ki67 (> 40%)**	206 (55.7%)	0.926	0.016	0.941	0.028	0.923	0.027	0.027	0.604
**CA125 (> 30.0 IU/mL)**	81 (21.9%)	2.306	< 0.001	2.378	< 0.001	2.405	< 0.001	0.001	0.750

The six patients who were falsely predicted to be low risk but actually had LNM all accepted chemotherapy and radiotherapy after surgery. All of them received regular follow-up in out-patient department. During a median follow-up of 38 months (24–78 months), no relapse was found in the six patients.

### The validation model

The performance of the prediction model was then validated in the validation cohort including 200 endometrial cancer patients from Shanghai Cancer Center. The AUC was 0.83 in validation cohort (95% CI, 0.75–0.92). A total of 119 patients (59.5%) were classified as being low risk. There were three patients (2.5%) with LNM in this low-risk group, and the negative predictive value was 97.5% (116/119). 23 patients (28.4%) out of the remaining 81 non-low-risk patients had LNM.

To further evaluate the predictive value of our model, we combined the prediction cohort and the validation cohort. The AUC was 0.83 among these 570 patients and the model predicted 348 patients as being at low risk. Nine patients in this low-risk group had LNM, and the negative predictive value was 97.4% (339/348). 25.2% (56/222) patients in the remaining non-low-risk patients had LNM.

We further divided the non-low-risk patients into two groups: an intermediate-risk group (predicted probability of LNM from 5% to 20%) and a high-risk group (predicted probability of LNM higher than 20%). The patients with one of the following conditions were classified as intermediate-risk: ①serum CA125 ≥ 30.0 IU/mL, PR staining > 50% and Ki67 < 40%; ②tumors with positive PR staining ≤ 50% and Ki67 ≥ 40%, CA125 < 30.0 IU/mL. This intermediate-risk group had 12.5% patients (15/120) with LNM, which is similar to that of the total population in this study. The patients with the following conditions were classified as high risk: serum CA125 ≥ 30.0 IU/mL, with PR staining ≤ 50% and/or Ki67 ≥ 40%. Among the 102 women with a high risk of LNM as predicted by the model, 41 (40.2%) had LNM.

### Comparison of prediction models

In order to evaluate the predictive efficiency of our model, we compared our model with three previously reported prediction models using the data from 370 patients from Ob&Gyn Hospital. We did not use the data from the 200 patients from Shanghai Cancer Center because the MRI data were not available in most of the patients. Because the purpose of this study was to identify the low-risk group, we compared the negative likelihood ratio and the negative post-test probability (PTP) between our model and other reported models. The negative PTP could be calculated at the 10% level of assumed prevalence of LNM using Bayes’ theorem.

As shown in Table 5, model A uses intraoperative frozen pathological grading, myometrial invasion, and the tumor size to estimate LNM. Among our 330 patients with available intra-operative frozen section diagnosis, 33.9% (112/330) were predicted as being at low risk. In this group, four patients (3.6%) had LNM. The negative likelihood ratio and negative PTP were 0.34 and 4% respectively.

**Table 5:** Comparison of predictive performance in different prediction models.

Model	Proportion of low-risk group	Number of LNM in low-risk group	Sensitivity (%)	Specificity (%)	Negative predictive value (%)	Negative likelihood ratio	Negative post-test probability (%)
**Our model**	61.9%	6	84.6	67.4	97.3	0.23	2
	(229/370)		(80.9–88.3)	(63.0–76.3)	(95.8–99.0)	(0.11–0.48)	(1–5)
**Model A**	38.1%**	4	87.5	36.2**	96.4	0.34	4
	(112/330)		(83.9–91.1)	(31.1–41.1)	(94.4–98.4)	(0.14–0.87)	(1–10)
**Model B**	65.4%	2	85.7	69.6	98.3	0.21	2
	(119/182)		(80.6–90.8)	(63.0–76.3)	(96.5–100.2)	(0.04–0.65)	(0.4–7)
**Model C**	85.1%**	25**	35.9*	87.6**	92.1*	0.73**	8*
	(315/370)		(31.0–40.8)	(84.3–97.0)	(89.3–94.8)	(0.58–0.93)	(6–9)
**Model D**	26.7%**	1	96.9	29.2**	98.9	0.11	1
	(88/330)		(95.0–98.8)	(24.3–34.1)	(97.7–100.0)	(0.02–0.74)	(0.2–8)
**Model E**	52.7%*	1	92.9	56.6*	99.0	0.13	1
	(96/182)		(89.1–96.7)	(49.4–63.8)	(97.5–100.4)	(0.02–0.84)	(0.2–9)

*: P < 0.05

**: P < 0.01

The predictive performance in each model was compared with our model.

Model D: model A combined with our model; Model E: model B combined with our model.

Model B predicts LNM by preoperative serum CA125 and three MRI parameters (deep myometrial invasion, extension beyond the corpus, and enlarged lymph nodes). Among our 182 patients with MRI data, 65.4% (119/182) were predicted as being at low risk. Two patients (1.6%) among them had LNM. The negative likelihood ratio and negative PTP were 0.21 and 2% respectively.

Model C estimated nodal status by analyzing postoperative pathological grade, myometrial invasion, LVSI, ER and PR. The model classified 85.1% (315/370) of patients as being at low risk. In this low-risk group, 25 patients (7.9%) had LNM. The negative likelihood ratio and negative PTP were 0.73 and 8% respectively.

Our model classified 61.9% (229/370) of the study population as being at low risk, 6 (2.6%) out of which had LNM. The negative likelihood ratio and negative PTP were 0.29 and 2% respectively. There was no statistically significant difference in predictive parameters between our model and models A or B (p > 0.05). Statistically significant differences were found in sensitivity, specificity, negative likelihood ratio and negative PTP between our model and model C (p < 0.05). Model C classified more patients with LNM in the low-risk group.

We then evaluated the predictive efficiency using combination of our model with model A or model B. The results showed that the combination of our model with either model A (model D) or model B (model E) could increase the sensitivity but decrease the specificity. Less patients would be classified into low-risk group. Using model B in collaboration with our model seemed to have a better predictive performance.

## Discussion

Correctly identifying endometrial cancer patients with low risk of LNM is important both before and after surgery. Deciding the patient being at low risk of LNM can prevent the patient from receiving lymphadenectomy of which the risk outweighs benefits for low risk patients. Predicting the status of LNM in endometrial cancer patients who received hysterectomy only without lymphadenectomy also helps the doctors to decide whether auxiliary radiotherapy or further lymphadenectomy should be applied or not.

Our study suggests that serum CA125 and the immunohistochemical markers PR and Ki67 can be used as reliable predictive factors for LNM in endometrioid endometrial cancer patients. With lower serum CA125 levels (< 30.0 IU/mL), tumor with either or both of higher expression of PR (> 50%) and lower expression of Ki67 (< 40%), the risk of LNM in a certain patient may be less than 4%.

We did not apply tumor grade in our prediction model because of the inconsistence of this pathologic diagnosis [18]. It is reported that the preoperative grade is upgraded in 15–20% of cases on final histology, and up to 24% of patients may be upstaged on final pathologic examination of grade or myometrial invasion [19,20].

Because correct scoring of the immunohistochemical markers is the key for our predicting model, it is of utmost importance to keep the immunostaining and evaluating processes standardized. Although there was no case in our group of which the difference of immunostaining score between the two observers exceeds 20%, we suggest the following procedures be done to ensure the quality of immunohistochemical scoring: (1)standardize the production process of samples; (2)ensure strict immunohistochemical negative controls; (3)observe the invasive portion of the most active tumor areas; (4)a senior pathologist or a group discussion is suggested if the score of immunohistochemical staining of the markers differed more than 20% between the two observers. With standard process and calibration between pathologists, immunohistochemical markers combined with serum CA125 are somehow objective, clinically available, and inexpensive.

There are several well performed prediction models using various parameters including serum CA125, MRI, tumor size, pathological grade, etc. Baak et al also reported the endometrial carcinoma prognostic index (ECPI) combining myometrium invasion, flow cytometric DNA ploidy, and morphometric mean shortest nuclear axis [MSNA] as good prognostic system in stage 1 and stage 2 endometrial carcinoma. Our prediction model demonstrated similar predictive performance to that of model A (intraoperative frozen pathological grading, myometrial invasion, and the tumor size) and model B (preoperative serum CA125 and three MRI parameters). This suggests that our prediction model could provide doctors one more choices in case certain parameters needed in other models were not available.

Furthermore, we evaluated the predictive performance using our model in collaboration with Model A or Model B. Our results showed that although the collaboration of these models could increase the sensitivity, the specificity was compromised. More patients without LNM would be classified into the non-low risk group. These results suggest that using one of these models alone is enough for predicting LNM, and combination of these models is not necessary.

In this study, we used immunohistochemical markers examined in postoperative samples. We suggest that immunohistochemical markers in preoperative endometrial biopsy specimens might also be used in predicting LNM preoperatively. Further prospective study should be performed to evaluate the consistency of immunohistochemical markers in endometrial biopsy sample and endometrial sample after hysterectomy.

Our study also has several limitations. Only 21.1% of our study population (120 of 570) underwent a systemic para-aortic lymphadenectomy. Recent studies [21,22] indicate that 3.5–4.0% of patients with endometrial cancer showed isolated para-aortic metastasis and had a worse prognosis. The incidence of isolated para-aortic metastasis in our study was 2.5% (3 of 120), and one of the three patients was classified as being at low risk. There is a chance that our low-risk model might have underestimated occult para-aortic metastasis.

In conclusion, we developed a possible prediction model for LNM using serum CA125 and immunohistochemical markers PR and Ki67. This model could provide doctors one more choice for predicting LNM in endometrial endometrioid cancer patients.
